# Molecular Epidemiology for Vector Research on Leishmaniasis

**DOI:** 10.3390/ijerph7030814

**Published:** 2010-03-05

**Authors:** Hirotomo Kato, Eduardo A Gomez, Abraham G Cáceres, Hiroshi Uezato, Tatsuyuki Mimori, Yoshihisa Hashiguchi

**Affiliations:** 1 Department of Veterinary Hygiene, Faculty of Agriculture, Yamaguchi University, Yamaguchi 753-8515, Japan; 2 Departamento de Oncocercosis, Servicio Nacional de Erradicacion de la Malaria, Ministerio de Salud Publica, Guayaquil 10833, Ecuador; E-Mail: egolandires@yahoo.es; 3 Sección de Entomología, Instituto de Medicina Tropical “Daniel A. Carrion”, Facultad de Medicina, Universidad Nacional Mayor de San Marcos, Lima, Lima 1, Perú; E-Mail: acaceres31@hotmail.com; 4 Laboratorio de Entomología, Instituto Nacional de Salud, Lima, Lima 11, Perú; 5 Department of Dermatology, Faculty of Medicine, University of the Ryukyus, Okinawa 903-0125, Japan; E-Mail: huezato@med.u-ryukyu.ac.jp; 6 Department of Microbiology, School of Health Sciences, Kumamoto University, Kumamoto 862-0976, Japan; E-Mail: mimori@kumamoto-u.ac.jp; 7 Department of Parasitology, Kochi Medical School, Kochi University, Kochi 783-8505, Japan; E-Mail: yhashiguchi42@yahoo.co.jp

**Keywords:** *Leishmania*, sand fly, epidemiology, mass screening

## Abstract

Leishmaniasis is a protozoan disease caused by the genus *Leishmania* transmitted by female phlebotomine sand flies. Surveillance of the prevalence of *Leishmania* and responsive vector species in endemic and surrounding areas is important for predicting the risk and expansion of the disease. Molecular biological methods are now widely applied to epidemiological studies of infectious diseases including leishmaniasis. These techniques are used to detect natural infections of sand fly vectors with *Leishmania* protozoa and are becoming powerful tools due to their sensitivity and specificity. Recently, genetic analyses have been performed on sand fly species and genotyping using PCR-RFLP has been applied to the sand fly taxonomy. In addition, a molecular mass screening method has been established that enables both sand fly species and natural leishmanial infections to be identified simultaneously in hundreds of sand flies with limited effort. This paper reviews recent advances in the study of sand flies, vectors of leishmaniasis, using molecular biological approaches.

## 1. Leishmaniasis

Leishmaniasis is caused by protozoan parasites belonging to the genus *Leishmania*, which is further divided into two subgenera, *Leishmania* and *Viannia. Leishmania* protozoa are transmitted by the bite of the female phlebotomine sand fly [1,2]. The disease is widely distributed around the world especially in tropical and subtropical areas, affecting at least 12 million people in 88 countries, with another 350 million people at risk [1,2]. Approximately 20 *Leishmania* species are known to be pathogenic to humans, and the species is the major determinant of clinical outcome (cutaneous, mucocutaneous and visceral forms) [1–3].

Cutaneous leishmaniasis (CL), known as “Oriental Sores” and “Baghdad Boils” in the Old World and “Chiclero’s Ulcer” and “Uta” in the New World, is caused by various *Leishmania* species including *Leishmania (Leishmania) major* and *L. (L.) tropica* in the Old World and *L. (L.) mexicana, L. (L.) amazonensis, L. (Viannia) braziliensis, L. (V.) guyanensis* and *L. (V.) peruviana* in the New World [1,2,4]. Typical CL is characterized by localized refractory skin ulcers or nodules at sites of infection that heal spontaneously leaving life-long scars [1,2]. Other unusual types of CL include leishmaniasis recidiva cutis (LRC) characterized by the development of satellite nodules in or around the scar of a clinically healed lesion after a period of time, diffuse cutaneous leishmaniasis (DCL) producing non-ulcerating chronic nodules over the entire body resembling skin lesions of lepromatous leprosy, and post kala-azar dermal leishmaniasis (PKDL), which can appear on the skin of individuals who have recovered from visceral leishmaniasis (VL) [1,2]. Some 90% of CL cases are reported to occur in just seven countries; Afghanistan, Algeria, Iran, Saudi Arabia, Syria, Brazil and Peru [2].

Mucocutaneous leishmaniasis (MCL), also known as “Espundia”, is endemic in Central and South America, and characterized by destructive metastatic lesions in the mucous membranes of the nose, mouth and throat cavities and surrounding tissues that occur months or years after the onset of the primary cutaneous infection [5]. Although the principal causative agent of MCL is *L. (V.) braziliensis*, other *Leishmania* species such as *L. (V.) guyanensis, L. (V.) panamensis* and *L. (L.) amazonensis* have been reported to affect mucosal tissues [6–8].

VL, caused by *L. (L.) donovani* complex, is also known as “Kala azar” or “Dum dum fever”. VL is the most severe form of leishmaniasis, and the clinical symptoms include irregular bouts of fever, substantial weight loss, fatigue, anemia and substantial swelling of the liver and spleen [9]. The disease is usually fatal if untreated, and is the second-largest parasitic killer in the world with an estimated 500,000 new cases and more than 50,000 deaths each year [2]. Some 90% of VL cases occur in just five countries; Bangladesh, India, Nepal, Sudan and Brazil [2].

**Figure 1.**
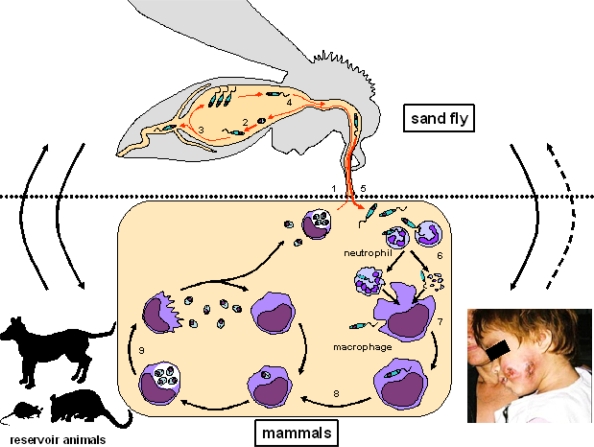
Schematic life cycle of *Leishmania* parasites. 1. The sand fly ingests amastigotes during blood feeding. 2. Amastigotes transform into promastigotes. 3. Promastigotes colonize and multiply at the hindgut and midgut of the sand fly. 4. Infective promastigotes (metacyclics) migrate to the anterior part of the gut. 5. The infective stage of promastigotes are transmitted to a mammalian host by the bites of the sand fly. 6. Promastigotes invade host neutrophils. 7. Macrophages are infected by promastigotes directly or through the phagocytosis of infected neutrophils, or infected silently by promastigotes released from apoptotic neutrophils. 8. Promastigotes transform into amastigotes. 9. Amastigotes multiply in infected cells by binary fission. Note: Most *Leishmania* species are maintained by an animal-to-animal transmission cycle and humans are considered an accidental host (arrow). However, anthroponotic transmission without animal reservoirs is also reported in some *Leishmania* species (dashed arrow).

Most leishmaniases are zoonotic diseases that include animal reservoir hosts in the transmission cycle [1]. Humans are considered to be an accidental host, although anthroponotic transmission without animal reservoirs is reported in some *Leishmania* species [1] (Figure 1).

The life cycle of *Leishmania* species involves two stages; in mammalian hosts, the parasites are observed as an intracellular amastigote with a round or ovoid-shaped and immotile form, but when taken up into the gut of sand flies, they become an extracellular promastigote characterized by a spindle-shaped and motile form with an external flagellum [10]. The transformation is triggered by a change in conditions such as temperature and pH [11,12]. The main target of the parasite in mammalian hosts is macrophages, and infections are known to occur in three ways; (1) direct infection, (2) phagocytosis of infected neutrophils by macrophages (the Trojan horse model), and 3) silent infection by parasites released from apoptotic neutrophils [13]. Neutrophils, which are recruited to sites of tissue damage caused by sand fly bites in the initial phase, are considered to ingest the majority of parasites and play a central role in the establishment of the *Leishmania* infection [13–18] (Figure 1).

## 2. Phlebotomine Sand Flies as a Vector of Leishmaniasis

**Figure 2.**
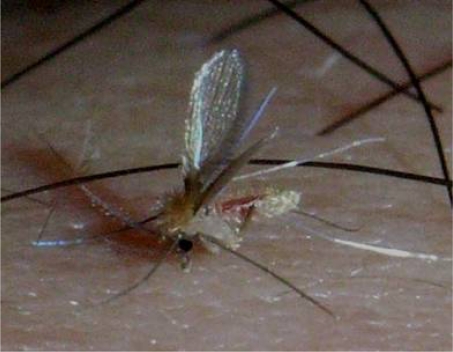
A blood-sucking phlebotomine sand fly.

Phlebotomine sand flies are tiny blood-feeding insects of the family Psychodidae in the order Diptera with a body length of approximately 2–3 mm [19]. They vary in color from silver-gray to almost black, and fold their wings into a characteristic V-shape when at rest (Figure 2). They are active nocturnally and rest in houses, cellars, caves and gaps among rocks in the daytime. Unlike mosquitoes, they fly feebly and silently, and sneak up on a host using a peculiar hopping motion [20]. Only female flies suck blood for egg production, and the bites are typically painful. To date, approximately 800 sand fly species have been recorded in five major genera; *Phlebotomus* (94 species) and *Sergentomyia* (258 species) in the Old World, and *Lutzomyia* (379 species), *Brumptomyia* (23 species) and *Warileya* (5 species) in the New World, and of these, proven vector species of *Leishmania* protozoa are classified into the genus *Phlebotomus* and *Lutzomyia* [20]. The majority of the species play no part in the transmission of leishmaniasis in nature for several reasons; they may not feed on blood from humans and potential reservoir animals, and/or they may be incapable of supporting the development of *Leishmania* species [20,21]. Less than 10% of sand flies have been incriminated as vector species of leishmaniasis, and only about 30 species have been demonstrated to have a vectorial capacity [10]. In addition, each vector species can only support the development of and consequently transmit certain species of *Leishmania* [10,19]. In the susceptible vector, the promastigotes of *Leishmania* attach to the epithelium of the gut, multiply, and differentiate into the infective metacyclic form, which is transmitted to the mammalian host [21,22] (Figure 1). The attachment of the promastigotes to the insect midgut is crucial to the completion of their life cycle in order to avoid excretion when the sand fly defecates [21,22]. The attachment is mediated by lipophosphoglycan (LPG), the major surface glycoconjugate of promastigotes, and the structures are polymorphic among species, suggesting that LPG is the major determinant specifying a vector species [22,23]. The only midgut protein of sand flies shown to interact with *Leishmania* is PpGalec, a β-galactoside-binding lectin, found in *Phlebotomus (P.) papatasi*, a principal vector of *L. (L.) major* in the Old World [24]. Genomic DNA hybridized with *PpGalec* was present in *P. papatasi* and *P. duboscqi*, both of which transmit *L. (L.) major* in nature, but absent in *P. sergenti* and *P. argentipes*, vector species of *L. (L.) tropica* and *L. (L.) donovani*, respectively, in the Old World, and *Lutzomyia* (*Lu.) longipalpis* and *Lu. verrucarum* which transmit *L. (L.) infantum* (previously called *L. (L.) chagasi*) and *L.(V.) peruviana*, respectively, in the New World [24]. The result strongly suggests that midgut molecules of sand flies are the key determinant of vectorial competency.

## 3. Advances in Sand Fly Taxonomy

As described, only some of the approximately 800 sand fly species are medically important, and certain sand fly species can transmit only certain species of *Leishmania* [21,22]. Since the spread of leishmaniases largely depends on the distribution of the vectors, the identification of circulating sand fly species in endemic and surrounding areas is important for predictions of the risk and expansion of the disease. Sand flies are generally identified as adults based on morphologic characteristics, mainly internal structures such as the spermatheca, cibarium and pharynx in females, and terminal genitalia in males [19,20]. Other characteristics include the location and intensity of pigmentation of the thorax, and the length ratios of wing veins, and antennal and leg segments [19,20]. Approximately 90 characteristics have been demonstrated as effective descriptors, and these characters are examined and measured on each specimen under a microscope after appropriate preservation and mounting [19,20]. Thus, the morphological classification requires considerable skill as well as taxonomic expertise. In addition, the presence of intraspecific variation and cryptic species frequently complicates classifications based on morphological features [25]. Furthermore, damage caused by improper storage and mounting makes the process more difficult or can cause misidentification. Therefore, other characteristics like molecular markers have been explored for the development of simpler and more accurate ways to identify sand flies. Several genetic markers have been used to examine the systematics, relationships and evolution among sand fly species and for population analyses within species [26–52]. Most results of genetic analyses support the generally accepted classification based on morphological characteristics, however, discrepancies exist between the two classifications in several groupings, suggesting the necessity for careful reconsideration of the sand fly taxonomy [42,51,52]. With the accumulation of genetic data on sand flies, attempts have been made to establish methods of identification using simple techniques such as the PCR-restriction fragment length polymorphism (RFLP) analysis of 18S rRNA genes [33,45–47,50,51]. Although genetic diversity affecting RFLP-patterns was found in some species, the genotyping method was shown to be accurate and easy-to-use for the identification of sand fly species, requiring less expertise and with less risk of different interpretations among researchers than the conventional morphology-based classification [33,45–47,50,51]. It is important to note that this DNA-based technique doesn’t require special storage conditions for the specimens, and different methods of preservation such as drying and the use of 70% ethanol or liquid nitrogen did not affect the quality of the results [33]. In addition, damage to samples, which affects the morphologic classification in many cases, does not affect the PCR-RFLP analysis. To date, approximately 400 *Lutzomyia* and 100 *Phlebotomus* species have been recorded, and it is impossible to distinguish them all with a PCR-RFLP-based method. Usually, however, not many species coexist in an endemic area. Therefore, the PCR-RFLP-based method targeting several different genes will be a powerful tool for sand fly research as well as studying taxonomy in given leishmaniasis-endemic areas.

Interestingly, several studies suggested differences in vectorial capacity among populations within vector species such as mosquitoes [53,54]. The population structure has been analyzed in the Old World species *P. papatasi* and *P. sergenti* [38,39] and in the New World species *Lu. longipalpis* and recently *Lu. hartmanni* and *Lu. ayacuchensis* [25,52,55–60]. Heterogeneity of transcribed spacer (ITS) 2 sequences among populations was reported in *P. sergenti*, the main vector of *L. (L.) tropica*, and an association with vectorial capacity was hypothesized [38]. In addition, remarkable genetic variation among populations was also found in *Lu. longipalpis*, the principal vector of *L. (L.) infantum* (=*L. (L.) chagasi)*, on the basis of the mitochondria ND4 gene [55]. On the other hand, an absence of geographic distribution for both ITS2 and mitochondria ND4 genes was reported in *P. papatasi*, the principal vector of *L. (L.) major* [39], and for ITS1 and ITS2 genes in both *Lu. hartmanni* and *Lu. ayacuchensis*, which are the vector species of *L. (V.) panamensis* and *L. (L.) mexicana*, respectively [52]. The genetic heterogeneity described in *P. sergenti* and *Lu. longipalpis* may be associated with morphological variation that is observed in the two species but not in *Lu. hartmanni*, *Lu. ayacuchensi*s and *P. papatasi* [39,52,59]. More recently, a correlation between microsatellite markers and geographical origin was described in *P. papatasi*, suggesting multi-locus microsatellite typing to be effective for population analysis in sand flies [61]. Thus, further extensive study should disclose markers appropriate for population genetics in sand flies, resulting in the elucidation of vectorial capacity among population structures.

## 4. Advances in the Detection and Identification of *Leishmania* Species within Naturally Infected Sand Flies

**Figure 3.**
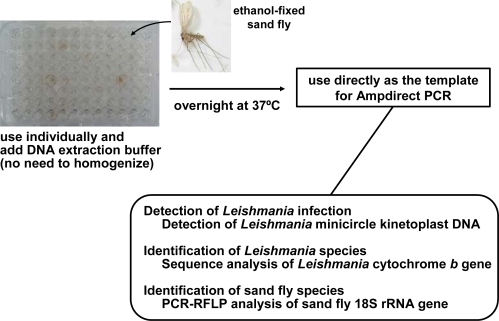
A molecular mass screening method for the detection of *Leishmania* within individual sand flies and identification of both sand fly and *Leishmania* species.

Detection and identification of *Leishmania* species within sand flies are important for predictions of the risk and expansion of the disease in endemic and surrounding areas since the infected species is the major determinant of the clinical outcome in humans [1–3]. The infection of sand flies with *Leishmania* promastigotes has been examined by the dissection of individual sand flies under a microscope. For this purpose, specimens need to be fresh, and the dissection of tiny sand flies requires a highly skilled technique. The procedure takes a relatively long time, and additionally, a large number of specimens have to be examined to obtain informative data for each area since the infection rate of sand flies with *Leishmania* is generally very low (0.01–1%) even in endemic areas [62,63]. To improve on conventional methods, several PCR-based techniques have been developed [45,47,64–73], and infection of *Leishmania* species within sand flies was identified, e.g., *L. (L.) major* in *P. papatasi* [65], *L. (L.) infantum* in *P. major* [69] and *L. (L.) donovani* in *P. argentipes* [72] in the Old World, and *L. (L.) mexicana* in *Lu. ayacuchensis* [45,47] and *Lu. ovallesi* [66], *L. (L.) amazonensis* in *Lu. longipalpis* [67,73], *L. (L.) infantum chagasi* in *Lu. longipalpis* [67] and *Lu. almerioi* [73], *L. peruviana* in *Lu. peruensis* [47], *L. (V.) naiffi* in *Lu. tortura* [48] and *L. (V.) braziliensis* in *Lu. ovallesi* [66], *Lu. gomezi* [66] and *Lu. neivai* [70] in the New World. In most studies, DNA was extracted from pooled sand fly samples or from individual sand flies by use of an expensive kit or by a complicated conventional protocol using proteinase K-containing DNA extraction buffer followed by phenol/chloroform extraction and ethanol precipitation. These methods are sensitive enough to detect *Leishmania* species within sand flies. However, several improvements were desirable for the analysis of a large number of sand flies with less effort and cost. In addition, it is better to analyze sand flies individually because several species co-exist in most endemic areas and the use of pooled samples may spoil important information on vector epidemiology such as the prevalent sand fly species and the relationships between *Leishmania* and vector species. Recently, a method of mass screening sand flies for *Leishmania* infection was established, and its usability for field research confirmed [46,47]. The protocol is represented in Figure 3. With this method, 96 individual samples can be analyzed at once with few processes. The method has a number of advantages: 1) purification of DNA is not required; that is, ethanol-fixed sand fly samples lysed in conventional DNA extraction buffer overnight at 37 °C without homogenization can be directly used as PCR templates, 2) the sensitivity and specificity are very high; leishmanial DNA is detectable if only one parasite exists in a sample, 3) data on individual sand flies can be obtained, 4) there is minimum risk of DNA loss and contamination among samples because of few processes, and 5) each sample can be used as a template for 100–150 PCRs. In addition, the sand fly species can be identified by molecular biological methods such as PCR-RFLP using the same template DNA if some genetic information is available on the prevalent sand fly species in the given endemic areas. Further, *Leishmania* species detected within sand flies can be identified by the analysis of leishmanial genes such as the mitochondrial cytochrome *b* gene [45–48,74–76]. Thus, application of the mass screening method in different areas will provide important information on risk factors, hopefully leading to the control and/or surveillance of leishmaniasis.

## 5. Concluding Remarks

Molecular biological techniques are now becoming powerful tools for sand fly research. Therefore, more detailed information on the risk factors for leishmaniasis, such as the prevalent sand fly species as well as the seasonal variation in the infection rate and transmission risk, can be accumulated by continuous efforts using such techniques in various endemic areas in different seasons. In addition, elucidation of the relationships between *Leishmania* and vector species, which requires enormous effort with current methods, by use of molecular methods mentioned, will contribute to not only epidemiological research on leishmaniasis but also basic studies on parasite-vector interactions.

## References

[1] Desjeux P The increase of risk factors for leishmaniasis worldwide Trans. R. Soc. Trop. Med. Hyg 2001 95 239 243 11490989 10.1016/s0035-9203(01)90223-8

[2] Desjeux P Leishmaniasis: current situation and new perspectives Comp. Immunol. Microbiol. Infect. Dis 2004 27 305 318 15225981 10.1016/j.cimid.2004.03.004

[3] Choi CM Lerner EA Leishmaniasis as an emerging infection J. Invest. Dermatol. Symp. Proc 2001 6 175 182 10.1046/j.0022-202x.2001.00038.x 11924824

[4] Grimaldi G Jr Tesh RB McMahon-Pratt D A review of the geographic distribution and epidemiology of leishmaniasis in the New World Am. J. Trop. Med. Hyg 1989 41 687 725 2701633 10.4269/ajtmh.1989.41.687

[5] Marsden PD Mucosal leishmaniasis (“espundia” Escomel, 1911) Trans. R. Soc. Trop. Med. Hyg 1986 80 859 876 3037735 10.1016/0035-9203(86)90243-9

[6] Naiff RD Talhari S Barrett TV Isolation of *Leishmania guyanensis* from lesions of the nasal mucosa Mem. Inst. Oswaldo Cruz 1988 83 529 530 3271946 10.1590/s0074-02761988000400022

[7] Barral A Pedral-Sampaio D Grimaldi G Jr Momen H McMahon-Pratt D Ribeiro de Jesus A Almeida R Badaro R Barral-Netto M Carvalho EM Johnson WD Jr Leishmaniasis in Bahia, Brazil: evidence that *Leishmania amazonensis* produces a wide spectrum of clinical disease Am. J. Trop. Med. Hyg 1991 44 536 546 2063957 10.4269/ajtmh.1991.44.536

[8] Osorio LE Castillo CM Ochoa MT Mucosal leishmaniasis due to *Leishmania (Viannia) panamensis* in Colombia: clinical characteristics Am. J. Trop. Med. Hyg 1998 59 49 52 9684627 10.4269/ajtmh.1998.59.49

[9] Bryceson ADM Leishmaniasis Manson’s Tropical Disease20th ed Cook CC W.B. Saunders Comp. Ltd London, Philadelphia, Tronto, Sydney and Tokyo 1996 1213 1245

[10] Bates PA Transmission of *Leishmania* metacyclic promastigotes by phlebotomine sand flies Int. J. Parasitol 2007 37 1097 1106 17517415 10.1016/j.ijpara.2007.04.003 PMC2675784

[11] Bates PA Rogers ME New insights into the developmental biology and transmission mechanisms of *Leishmania* Curr. Mol. Med 2004 4 601 609 15357211 10.2174/1566524043360285

[12] Kamhawi S Phlebotomine sand flies and *Leishmania* parasites: friends or foes? Trends Parasitol 2006 22 439 445 16843727 10.1016/j.pt.2006.06.012

[13] Peters NC Sacks DL The impact of vector-mediated neutrophil recruitment on cutaneous leishmaniasis Cell. Microbiol 2009 11 1290 1296 19545276 10.1111/j.1462-5822.2009.01348.x PMC3431610

[14] Laskay T van Zandbergen G Solbach W Neutrophil granulocytes—Trojan horses for *Leishmania major* and other intracellular microbes? Trends Microbiol 2003 11 210 214 12781523 10.1016/s0966-842x(03)00075-1

[15] Ribeiro-Gomes FL Otero AC Gomes NA Moniz-De-Souza MC Cysne-Finkelstein L Arnholdt AC Calich VL Coutinho SG Lopes MF DosReis GA Macrophage interactions with neutrophils regulate *Leishmania major* infection J. Immunol 2004 172 4454 4462 15034061 10.4049/jimmunol.172.7.4454

[16] van Zandbergen G Klinger M Mueller A Dannenberg S Gebert A Solbach W Laskay T Cutting edge: neutrophil granulocyte serves as a vector for *Leishmania* entry into macrophages J. Immunol 2004 173 6521 6525 15557140 10.4049/jimmunol.173.11.6521

[17] Peters NC Egen JG Secundino N Debrabant A Kimblin N Kamhawi S Lawyer P Fay MP Germain RN Sacks D *In vivo* imaging reveals an essential role for neutrophils in leishmaniasis transmitted by sand flies Science 2008 321 970 974 18703742 10.1126/science.1159194 PMC2606057

[18] Jochim RC Teixeira C *Leishmania* commandeers the host inflammatory response through neutrophils Trends Parasitol 2009 25 145 147 19269250 10.1016/j.pt.2009.01.001

[19] Young DG Duncan MAGuide to the identification and geographic distribution of *Lutzomyia* sand flies in Mexico, the West Indies, Central and South America (Diptera: Psychodidae), Memoirs of the American Entomological Institute, 54, Associated Publishers—American Entomological Institute, Gainsville, FL, USA, 1994

[20] Munstermann LE Phlebotomine sand flies, the Psychodidae Biology of Disease Vectors2nd ed Marquardt WC Black WC Freier JE Hagedorn HH Hemingway J Higgs S James AA Kondratieff B Moore CG Elsevier San Diego, CA, USA 2004 141 151

[21] Killick-Kendrick R The biology and control of phlebotomine sand flies Clin. Dermatol 1999 17 279 289 10384867 10.1016/s0738-081x(99)00046-2

[22] Sacks DL *Leishmania*-sand fly interactions controlling species-specific vector competence Cell. Microbiol 2001 3 189 196 11298643 10.1046/j.1462-5822.2001.00115.x

[23] Sacks DL Modi G Rowton E Späth G Epstein L Turco SJ Beverley SM The role of phosphoglycans in *Leishmania*-sand fly interactions Proc. Natl. Acad. Sci. USA 2000 97 406 411 10618431 10.1073/pnas.97.1.406 PMC26676

[24] Kamhawi S Ramalho-Ortigao M Pham VM Kumar S Lawyer PG Turco SJ Barillas-Mury C Sacks DL Valenzuela JG A role for insect galectins in parasite survival Cell 2004 119 329 341 15543683 10.1016/j.cell.2004.10.009

[25] Bauzer LG Souza NA Maingon RD Peixoto AA *Lutzomyia longipalpis* in Brazil: a complex or a single species? A mini-review Mem. Inst. Oswaldo Cruz 2007 102 1 12 17293992 10.1590/s0074-02762007000100001

[26] Ready PD Smith DF Killick-Kendrick R DNA hybridizations on squash-blotted sandflies to identify both *Phlebotomus papatasi* and infecting *Leishmania major* Med. Vet. Entomol 1988 2 109 116 2856540 10.1111/j.1365-2915.1988.tb00060.x

[27] Ready PD Lainson R Shaw JJ Souza AA DNA probes for distinguishing *Psychodopygus wellcomei* from *Psychodopygus* complexus (Diptera:Psychodidae) Mem. Inst. Oswaldo Cruz 1991 86 41 49 1842400 10.1590/s0074-02761991000100008

[28] Ready PD de Souza AA Rebelo JM Day JC Silveira FT Campbell-Lendrum D Davies CR Costa JM Phylogenetic species and domesticity of *Lutzomyia whitmani* at the southeast boundary of Amazonian Brazil Trans. R. Soc. Trop. Med. Hyg 1998 92 159 160 9764319 10.1016/s0035-9203(98)90726-x

[29] Booth DR Ready PD Smith DF Retrotransposons and evolution in phlebotomines Parassitologia 1991 33 105 112 1726736

[30] Booth DR Ready PD Smith DF Isolation of non-LTR retrotransposon reverse transcriptase-like sequences from phlebotomine sandflies Insect Mol. Biol 1994 3 89 96 7527283 10.1111/j.1365-2583.1994.tb00155.x

[31] Booth DR Ready PD Smith DF Evolution of multiple families of non-LTR retrotransposons in phlebotomine sandflies Genet. Res 1996 67 227 237 8690271 10.1017/s0016672300033711

[32] Esseghir S Ready PD Killick-Kendrick R Ben-Ismail R Mitochondrial haplotypes and phylogeography of *Phlebotomus* vectors of *Leishmania major* Insect Mol. Biol 1997 6 211 225 9272439 10.1046/j.1365-2583.1997.00175.x

[33] Aransay AM Scoulica E Chaniotis B Tselentis Y Typing of sand flies from Greece and Cyprus by DNA polymorphism of 18S rRNA gene Insect Mol. Biol 1999 8 179 184 10380101 10.1046/j.1365-2583.1999.820179.x

[34] Aransay AM Scoulica E Tselentis Y Ready PD Phylogenetic relationships of phlebotomine sandflies inferred from small subunit nuclear ribosomal DNA Insect Mol. Biol 2000 9 157 168 10762423 10.1046/j.1365-2583.2000.00168.x

[35] Aransay AM Ready PD Morillas-Marquez F Population differentiation of *Phlebotomus perniciosus* in Spain following postglacial dispersal Heredity 2003 90 316 325 12692585 10.1038/sj.hdy.6800246

[36] Campbell-Lendrum DH Brandão-Filho SP Pinto MC Vexenat A Ready PD Davies CR Domesticity of *Lutzomyia whitmani* (Diptera: psychodidae) populations: field experiments indicate behavioural differences Bull. Entomol. Res 2000 90 41 48 10948362

[37] Di Muccio T Marinucci M Frusteri L Maroli M Pesson B Gramiccia M Phylogenetic analysis of *Phlebotomus* species belonging to the subgenus *Larroussius* (Diptera, psychodidae) by ITS2 rDNA sequences Insect Biochem. Mol. Biol 2000 30 387 393 10745162 10.1016/s0965-1748(00)00012-6

[38] Depaquit J Ferté H Léger N Lefranc F Alves-Pires C Hanafi H Maroli M Morillas-Marquez F Rioux JA Svobodova M Volf, P. ITS 2 sequences heterogeneity in *Phlebotomus sergenti* and *Phlebotomus similis* (Diptera, Psychodidae): possible consequences in their ability to transmit *Leishmania tropica* Int. J. Parasitol 2002 32 1123 1131 12117495 10.1016/s0020-7519(02)00088-7

[39] Depaquit J Lienard E Verzeaux-Griffon A Ferté H Bounamous A Gantier JC Hanafi HA Jacobson RL Maroli M Moin-Vaziri V Müller F Ozbel Y Svobodova M Volf P Léger N Molecular homogeneity in diverse geographical populations of *Phlebotomus papatasi* (Diptera, Psychodidae) inferred from ND4 mtDNA and ITS2 rDNA epidemiological consequences Infect. Genet. Evol 2008 8 159 170 18243814 10.1016/j.meegid.2007.12.001

[40] Testa JM Montoya-Lerma J Cadena H Oviedo M Ready PD Molecular identification of vectors of *Leishmania* in Colombia: mitochondrial introgression in the *Lutzomyia townsendi* series Acta Trop 2002 84 205 218 12443799 10.1016/s0001-706x(02)00187-0

[41] Torgerson DG Lampo M Velazquez Y Woo PT Genetic relationships among some species groups within the genus *Lutzomyia* (Diptera: Psychodidae) Am. J. Trop. Med. Hyg 2003 69 484 493 14695085

[42] Beati L Caceres AG Lee JA Munstermann LE Systematic relationships among *Lutzomyia* sand flies (Diptera: Psychodidae) of Peru and Colombia based on the analysis of 12S and 28S ribosomal DNA sequences Int. J. Parasitol 2004 34 225 234 15037108 10.1016/j.ijpara.2003.10.012

[43] Yahia H Ready PD Hamdani A Testa JM Guessous-Idrissi N Regional genetic differentiation of *Phlebotomus sergenti* in three Moroccan foci of cutaneous leishmaniasis caused by *Leishmania tropica* Parasite 2004 11 189 199 15224581 10.1051/parasite/2004112189

[44] Pesson B Ready JS Benabdennbi I Martín-Sánchez J Esseghir S Cadi-Soussi M Morillas-Marquez F Ready PD Sandflies of the *Phlebotomus perniciosus* complex: mitochondrial introgression and a new sibling species of *P. longicuspis* in the Moroccan Rif Med. Vet. Entomol 2004 18 25 37 15009443 10.1111/j.0269-283x.2004.0471.x

[45] Kato H Uezato H Katakura K Calvopiña M Marco JD Barroso PA Gomez EA Mimori T Korenaga M Iwata H Nonaka S Hashiguchi Y Detection and identification of *Leishmania* species within naturally infected sand flies in the Andean areas of Ecuador by a polymerase chain reaction Am. J. Trop. Med. Hyg 2005 72 87 93 15728872

[46] Kato H Uezato H Gomez EA Terayama Y Calvopiña M Iwata H Hashiguchi Y Establishment of a mass screening method of sand fly vectors for *Leishmania* infection by molecular biological methods Am. J. Trop. Med. Hyg 2007 77 324 329 17690406

[47] Kato H Cáceres AG Gomez EA Mimori T Uezato H Marco JD Barroso PA Iwata H Hashiguchi Y Molecular mass screening to incriminate sand fly vectors of Andean-type cutaneous leishmaniasis in Ecuador and Peru Am. J. Trop. Med. Hyg 2008 79 719 721 18981511

[48] Kato H Gomez EA Yamamoto Y Calvopiña M Guevara AG Marco JD Barroso PA Iwata H Hashiguchi Y Natural infection of *Lutzomyia tortura* with *Leishmania (Viannia) naiffi* in an Amazonian area of Ecuador Am. J. Trop. Med. Hyg 2008 79 438 440 18784239

[49] Barón S Martín-Sánchez J Gállego M Morales-Yuste M Boussaa S Morillas-Márquez F Intraspecific variability (rDNA ITS and mtDNA Cyt b) of *Phlebotomus sergenti* in Spain and Morocco Acta Trop 2008 107 259 267 18703008 10.1016/j.actatropica.2008.07.003

[50] Barroso PA Marco JD Kato H Tarama R Rueda P Cajal SP Basombrío MA Korenaga M Taranto NJ Hashiguchi Y The identification of sand fly species, from an area of Argentina with endemic leishmaniasis, by the PCR-based analysis of the gene coding for 18S ribosomal RNA Ann. Trop. Med. Parasitol 2007 101 247 253 17362599 10.1179/136485907X156988

[51] Terayama Y Kato H Gomez EAL Uezato H Calvopiña M Iwata H Hashiguchi Y Molecular typing of sand fly species (Diptera, Psychodidae, Phlebotominae) from areas endemic for leishmaniasis in Ecuador by PCR-RFLP of 18S ribosomal RNA gene J. Vet. Med. Sci 2008 70 907 913 18840964 10.1292/jvms.70.907

[52] Kuwahara K Kato H Gomez EA Uezato H Mimori T Yamamoto YI Calvopiña M Cáceres AG Iwata H Hashiguchi Y Genetic diversity of ribosomal RNA internal transcribed spacer sequences in *Lutzomyia* species from areas endemic for New World cutaneous leishmaniasis Acta Trop 2009 112 131 136 19631188 10.1016/j.actatropica.2009.07.010

[53] Krzywinski J Besansky NJ Molecular systematics of *Anopheles*: from subgenera to subpopulations Annu. Rev. Entomol 2003 48 111 139 12208816 10.1146/annurev.ento.48.091801.112647

[54] Obsomer V Defourny P Coosemans M The *Anopheles dirus* complex: spatial distribution and environmental drivers Malar. J 2007 6 26 17341297 10.1186/1475-2875-6-26 PMC1838916

[55] Soto SI Lehmann T Rowton ED Vélez BID Porter CH Speciation and population structure in the morphospecies *Lutzomyia longipalpis* (Lutz & Neiva) as derived from the mitochondrial ND4 gene Mol. Phylogenet. Evol 2001 18 84 93 11161745 10.1006/mpev.2000.0863

[56] Bottecchia M Oliveira SG Bauzer LG Souza NA Ward RD Garner KJ Kyriacou CP Peixoto AA Genetic divergence in the cacophony IVS6 intron among five Brazilian populations of *Lutzomyia longipalpis* J. Mol. Evol 2004 58 754 761 15461432 10.1007/s00239-004-2586-y

[57] Bauzer LG Souza NA Ward RD Kyriacou CP Peixoto AA The period gene and genetic differentiation between three Brazilian populations of *Lutzomyia longipalpis* Insect. Mol. Biol 2002 11 315 323 12144696 10.1046/j.1365-2583.2002.00340.x

[58] Bauzer LG Gesto JS Souza NA Ward RD Hamilton JG Kyriacou CP Peixoto AA Molecular divergence in the period gene between two putative sympatric species of the *Lutzomyia longipalpis* complex Mol. Biol. Evol 2002 19 1624 1627 12200489 10.1093/oxfordjournals.molbev.a004224

[59] Maingon RD Ward RD Hamilton JG Bauzer LG Peixoto AA The *Lutzomyia longipalpis* species complex: does population sub-structure matter to *Leishmania* transmission? Trends Parasitol 2008 24 2 7 10.1016/j.pt.2007.10.003 18023260

[60] Lins RM Souza NA Peixoto AA Genetic divergence between two sympatric species of the *Lutzomyia longipalpis* complex in the paralytic gene, a locus associated with insecticide resistance and lovesong production Mem. Inst. Oswaldo Cruz 2008 103 736 740 19057828 10.1590/s0074-02762008000700019

[61] Hamarsheh O Presber W Yaghoobi-Ershadi MR Amro A Al-Jawabreh A Sawalha S Al-Lahem A Das ML Guernaoui S Seridi N Dhiman RC Hashiguchi Y Ghrab J Hassan M Schönian G Population structure and geographical subdivision of the *Leishmania major* vector *Phlebotomus papatasi* as revealed by microsatellite variation Med. Vet. Entomol 2009 23 69 77 19239616 10.1111/j.1365-2915.2008.00784.x

[62] Hashiguchi Y Gomez EAL A review of leishmaniasis in Ecuador Bull. Pan Am. Hlth. Org 1991 25 64 76 2054554

[63] Hashiguchi Y Leishmaniasis Progress of Medical Parasitology in Japan Otsuru M Kamegai S Hayashi S Megro Parasitological Museum Tokyo, Japan 2003 7 537 553

[64] Aransay AM Scoulica E Tselentis Y Detection and identification of *Leishmania* DNA within naturally infected sand flies by seminested PCR on minicircle kinetoplast DNA Appl. Environ. Microbiol 2000 66 1933 1938 10788363 10.1128/aem.66.5.1933-1938.2000 PMC101436

[65] Parvizi P Mauricio I Aransay AM Miles MA Ready PD First detection of *Leishmania major* in peridomestic *Phlebotomus papatasi* from Isfahan province, Iran: comparison of nested PCR of nuclear ITS ribosomal DNA and semi-nested PCR of minicircle kinetoplast DNA Acta Trop 2005 93 75 83 15589800 10.1016/j.actatropica.2004.09.007

[66] Jorquera A González R Marchán-Marcano E Oviedo M Matos M Multiplex-PCR for detection of natural *Leishmania* infection in *Lutzomyia* spp. captured in an endemic region for cutaneous leishmaniasis in state of Sucre, Venezuela Mem. Inst. Oswaldo Cruz 2005 100 45 48 15867962 10.1590/s0074-02762005000100008

[67] Paiva BR Secundino NF Nascimento JC Pimenta PF Galati EA Junior HF Malafronte RS Detection and identification of *Leishmania* species in field-captured phlebotomine sandflies based on mini-exon gene PCR Acta Trop 2006 99 252 259 17055444 10.1016/j.actatropica.2006.08.009

[68] Córdoba-Lanús E De Grosso ML Piñero JE Valladares B Salomón OD Natural infection of *Lutzomyia neivai* with *Leishmania* spp. in northwestern Argentina Acta Trop 2006 98 1 5 16529708 10.1016/j.actatropica.2005.11.010

[69] Azizi K Rassi Y Javadian E Motazedian MH Asgari Q Yaghoobi-Ershadi MR First detection of *Leishmania infantum* in *Phlebotomus (Larroussius) major* (Diptera: Psychodidae) from Iran J. Med. Entomol 2008 45 726 731 18714874 10.1603/0022-2585(2008)45[726:fdolii]2.0.co;2

[70] Pita-Pereira D Souza GD Zwetsch A Alves CR Britto C Rangel EF First report of *Lutzomyia (Nyssomyia) neivai* (Diptera: Psychodidae: Phlebotominae) naturally infected by *Leishmania (Viannia) braziliensis* in a periurban area of south Brazil using a multiplex polymerase chain reaction assay Am. J. Trop. Med. Hyg 2009 80 593 595 19346382

[71] Silva EA Andreotti R Dias ES Barros JC Brazuna JC Detection of *Leishmania* DNA in phlebotomines captured in Campo Grande, Mato Grosso do Sul, Brazil Exp. Parasitol 2008 119 343 348 18456262 10.1016/j.exppara.2008.03.011

[72] Pandey K Pant S Kanbara H Shuaibu MN Mallik AK Pandey BD Kaneko O Yanagi T Molecular detection of *Leishmania* parasites from whole bodies of sandflies collected in Nepal Parasitol. Res 2008 103 293 297 18415124 10.1007/s00436-008-0967-7

[73] Savani ES Nunes VL Galati EA Castilho TM Zampieri RA Floeter-Winter LM The finding of *Lutzomyia almerioi* and *Lutzomyia longipalpis* naturally infected by *Leishmania* spp. in a cutaneous and canine visceral leishmaniases focus in Serra da Bodoquena, Brazil Vet. Parasitol 2009 160 18 24 19062193 10.1016/j.vetpar.2008.10.090

[74] Luyo-Acero G Uezato H Oshiro M Kariya K Katakura K Gomez EAL Hashiguchi Y Nonaka S Sequence variation of the cytochrome *b* gene of various human infecting members of the genus *Leishmania* and their pathology Parasitol 2004 128 483 491 10.1017/s0031182004004792 15180316

[75] Marco JD Bhutto AM Soomro FR Baloch JH Barroso PA Kato H Uezato H Katakura K Korenaga M Nonaka S Hashiguchi Y Multilocus enzyme electrophoresis and cytochrome *b* gene sequencing-based identification of *Leishmania* isolates from different foci of cutaneous leishmaniasis in Pakistan Am. J. Trop. Med. Hyg 2006 75 261 266 16896129

[76] Marco JD Uezato H Mimori T Barroso PA Korenaga M Nonaka S Basombrio MA Taranto NJ Hashiguchi Y Are cytochrome *b* gene sequencing and polymorphism-specific polymerase chain reaction as reliable as multilocus enzyme electrophoresis for identifying *Leishmania* spp. from Argentina? Am. J. Trop. Med. Hyg 2006 75 256 260 16896128

